# Potato Pathogens in Russia’s Regions: An Instrumental Survey with the Use of Real-Time PCR/RT-PCR in Matrix Format

**DOI:** 10.3390/pathogens8010018

**Published:** 2019-01-29

**Authors:** Alexander Malko, Pavel Frantsuzov, Maksim Nikitin, Natalia Statsyuk, Vitaly Dzhavakhiya, Alexander Golikov

**Affiliations:** 1 Russian Agricultural Center, Moscow 107139, Russia; alexmalko@mail.ru; 2 GenBit LLC, Nauchny pr. 20/2, Moscow 117246, Russia; frantsuzov@genbitgroup.com (P.F.); nikitin@genbitgroup.com (M.N.); golikov@genbitgroup.com (A.G.); 3 All-Russian Research Institute of Phytopathology, Bolshie Vyazemy 143050, Russia; dzhavakhiya@yahoo.com

**Keywords:** real-time PCR, potato viral diseases, potato bacterial diseases, high-throughput diagnostics, matrix approach, multiplex pathogen identification, occurrence of pathogens

## Abstract

Viral and bacterial diseases of potato cause significant yield loss worldwide. The current data on the occurrence of these diseases in Russia do not provide comprehensive understanding of the phytosanitary situation. Diagnostic systems based on disposable stationary open qPCR micromatrices intended for the detection of eight viral and seven bacterial/oomycetal potato diseases have been used for wide-scale screening of target pathogens to estimate their occurrence in 11 regions of Russia and to assess suitability of the technology for high-throughput diagnostics under conditions of field laboratories. Analysis of 1025 leaf and 725 tuber samples confirmed the earlier reported data on the dominance of potato viruses Y, S, and M in most regions of European Russia, as well as relatively high incidences of *Clavibacter michiganensis* subsp. *sepedonicus*, *Pectobacterium atrosepticum*, and *P. carotovorum* subsp. *carotovorum*, and provided detailed information on the phytosanitary status of selected regions and geographical spread of individual pathogens. Information on the occurrence of mixed infections, including their composition, was the first data set of this kind for Russia. The study is the first large-scale screening of a wide range of potato pathogens conducted in network mode using unified methodology and standardized qPCR micromatrices. The data represent valuable information for plant pathologists and potato producers and indicate the high potential of the combined use of matrix PCR technology and network approaches to data collection and analysis with the view to rapidly and accurately assess the prevalence of certain pathogens, as well as the phytosanitary state of large territories.

## 1. Introduction

Potato belongs to the world’s staple crops with the global production volume over 375 million tons [1]. Billions of people depend on this crop, so its sustainable production is very important for global food security. China, India, and Russia are principal potato producers [1]. However, crop capacity and quality of potatoes produced by these and many other countries are far behind those in developed countries. One of the reasons for that is the spread of pathogenic microorganisms posing a serious threat to potato industry. According to some data, the total annual potato loss due to bacteria/fungi and viruses worldwide is 14% and 7%, respectively [2].

Viral and viroid infections of potatoes are especially dangerous because of potential infection transmission via seed tubers due to the vegetative reproduction of the crop. Among more than 40 viruses and viroids infecting cultivated potatoes, eight (potato leaf roll virus (PLRV), potato virus Y (PVY), potato virus X (PVX), potato virus A (PVA), potato virus S (PVS), potato virus M (PVM), potato mop-top virus (PMTV), and potato spindle tuber viroid (PSTVd)) are economically the most significant in terms of their global distribution and potential negative effect. Single-virus infection may result in the yield loss reaching 80–90% for PVY and PLRV, 50% for PVX, and up to 20–40% for the other five viruses [3,4]. Mixed viral infection, which is common in fields, may have an even more severe impact [4,5,6]; thus, it has been reported that single-virus infection of potato with PVX and PVY caused yield losses of 16 and 23%, respectively, while co-infection with both viruses decreased yield by 39% [7] (cited by Reference [4]).

Another economically significant group of potato pathogens are bacteria; the most important are *Clavibacter michiganensis* subsp. *sepedonicus* (up to 50% of yield losses [8]), *Ralstonia solanacearum* (from 10–15% to 80–100% of yield losses [9]), and pectolytic bacteria causing soft rot and blackleg in potato: *Pectobacterium atrosepticum*, *P. carotovorum* subsp. *carotovorum*, and various *Dickeya* species (up to 25% yield reduction and 30% of storage losses [10]).

Unlike with fungal pathogens, once the symptoms of either viral or bacterial infection appear, there are no control methods available for these pathogens; then, only preventive methods may be applied. Therefore, the permanent monitoring of these pathogens appears crucial in large countries like Russia, whose territories cover different climatic zones. 

Unfortunately, there is no integrated monitoring system for major viral and bacterial potato diseases in Russia, and only a few pathogens or regions are referred to often in available studies [11,12,13,14,15,16,17,18]. 

A similar situation is also common in other countries. Reports are focused on the monitoring of one or two pathogens within the whole country (see, for example, References [19,20,21,22]), and only a few studies describe the screening of three or four viral or bacterial pathogens in certain areas or small countries [23,24]. As far as we know, detailed information about the presence of the major potato pathogens in the majority of potato-producing regions of a country was reported only for viruses in China [25,26], Tunisia [27], and Pakistan [28] by including information about the presence and composition of mixed viral infections [26,28].

**Figure 1:**
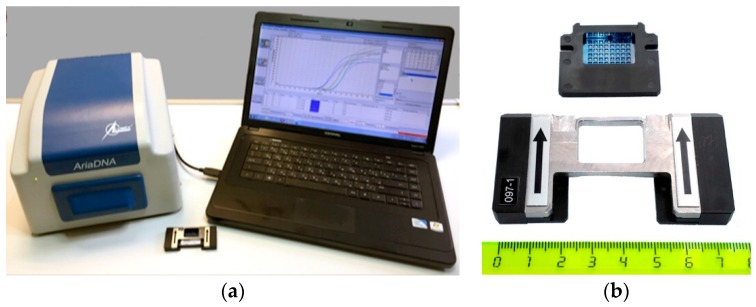
(**a**) AriaDNA® microchip amplifier with laptop and PCR micromatrix in a holder. (**b**) A 48-well micro-matrix and a holder (the numbers on the ruler represent centimeters).

To date, the majority of existing diagnostic methods is based on immunological and nucleic acid-based detection techniques; these approaches are permanently improved to achieve the possibility of a reliable high-throughput analysis [29,30]. Recently a diagnostic system based on stationary open PCR/RT-PCR micromatrices was developed, which provides a simultaneous detection and identification of a wide range of plant pathogens. The system includes a portable two-beam AriaDNA® microchip amplifier (Lumex-Marketing LLC, St.-Petersburg, Russia) intended to work in a real-time mode with disposable micromatrices consisting of 30 or 48 microreactors/wells (Figure 1). In all the wells, reactions are performed under the same “standard” amplification conditions. For RT-PCR (reverse transcription followed by PCR), both reactions are sequentially performed in the same well. An important feature of the technology is that all components of the PCR/RT-PCR master mix (including reverse transcriptase and polymerase) are immobilized and lyophilized in the wells; this reduces the time of analysis and the number of required manipulations, and provides a long (3 and 6 months for RT-PCR and PCR, respectively) shelf-life of ready-to-use micromatrices at room temperature that significantly simplifies their storage and transportation [31].

Earlier, we developed and validated test systems for two micromatrices intended for the detection of eight viruses and viroids (ordinary and necrotic forms of PVY, PVX, PVA, PVS, PVM, PLRV, PMTV, and PSTVd), and seven bacterial/oomycetal (*Pectobacterium atrosepticum*, *P. carotovorum* subsp. *carotovorum*, *Dickeya dianthicola*, *D. solani*, *Clavibacter michiganensis* subsp. *sepedonicus*, *Ralstonia solanacearum*, and *Phytophthora infestans*) pathogens of potato [32,33]. 

The purpose of this study was to conduct a large-scale survey of potato pathogens in field samples collected from different regions of Russia using AriaDNA® amplifiers and two above-mentioned micro-matrices in order to estimate the occurrence of the target pathogens in the chosen regions, as well as to assess the suitability of the technology for high-throughput screening of multiple pathogens of potato under the conditions of field laboratories.

## 2. Results

### 2.1. Bacterial/Oomycetal Infections

Results of the survey of target infections across the studied regions are shown in Table 1. The lowest level of infestation (7.9%) with target pathogens was observed in the Moscow region (3 out of 38 samples), while the highest infection level (64.5%) was revealed in Tver’ region (40 out of 62 samples).

**Figure 2:**
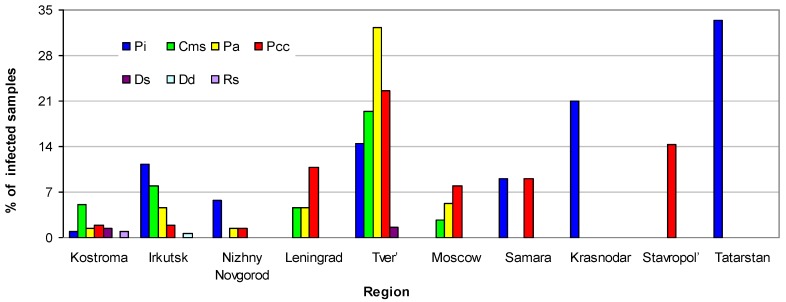
Frequency of the target DNA-based pathogens of potato in different regions. Rs, *Ralstonia solanacearum*; Pa, *Pectobacterium atrosepticum*; Pcc, *P. carotovorum* subsp. *carotovorum*; Cms, *Clavibacte rmichiganensis* subsp. *sepedonicus*; Pi, *Phytophthora infestans*; Ds, *Dickeya solani*, Dd, *D. dianthicola*.

The maximum diversity of DNA-based pathogens was observed in Kostroma, Tver’, and Irkutsk regions (6, 5, and 5 pathogens, respectively; Figure 2). The widest geographical spread (8 of 10 regions) was registered for *P. carotovorum* subsp. *carotovorum*. At the same time, only one sample infected with *D. dianthicola* was found. Two samples from Kostroma region contained *R. solanacearum* belonging to the A2 list of quarantine objects in Russia, and 39 samples from five regions (Tver’, Irkutsk, Kostroma, Leningrad, and Moscow) were infected with *C. michiganensis* subsp. *sepedonicus*, which belongs to the list of regulated nonquarantine pathogens. 

**Figure 3:**
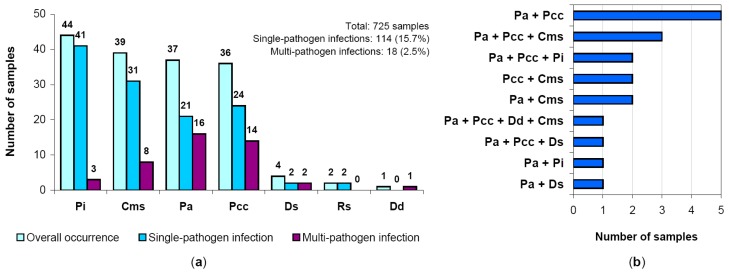
(**a**) Occurrence of single- and multi-pathogen bacterial/oomycetal infections in analyzed samples. (**b**) Details of multi-pathogen infections. Rs, *Ralstonia solanacearum*; Pa, *Pectobacterium atrosepticum*; Pcc, *P. carotovorum* subsp. *carotovorum*; Cms, *Clavibacter michiganensis* subsp. *sepedonicus*; Pi, *Phytophthora infestans*; Ds, *Dickeya solani*, Dd, *D. dianthicola*.

The occurrence of different pathogens in infected samples is shown in Figure 3a. The most frequent were *P. infestans*, *C. michiganensis* subsp. *sepedonicus*, *P. atrosepticum*, and *P. carotovorum* subsp. *carotovorum*. The percentage of these pathogens in the total pool of infected samples (with account of samples with multi-pathogen infections) was 33.3%, 29.5%, 28%, and 27.2%, respectively.

Mixed (multi-pathogen) infection was observed in 18 samples from five regions (Leningrad, Kostroma, Moscow, Tver’, and Irkutsk). Among them, there were five combinations of two pathogens, three combinations of three pathogens, and one sample contained four pathogens (Figure 3b). Domination of mixed infections causing potato blackleg disease was obvious. The most frequent components of mixed bacterial infections were *P. atrosepticum*, *P. carotovorum* subsp. *carotovorum*, and *C. michiganensis* subsp. *sepedonicus*, which were detected in 16, 14, and 8 samples with mixed infection, respectively; at the same time, *R. solanacearum* was the only pathogen not observed in multi-pathogen affected samples (Figure 3a,b).

### 2.2. Viral/Viroid Infections

Results of the survey of viral infection of potato across the studied regions are shown in Table 2. The most widely occurring viruses were PVY^O^, PVS, PVM, and PVY^NTN^ (nine regions each), while other viruses were observed in two regions each except PMTV, which was observed in three regions. The highest level of PVY^O^ presence was observed in Tver’ and Leningrad regions, as well as in the Republic of Tatarstan (25.0%, 18.1%, and 19.3% of the total number of samples, respectively). The highest level of PVS infection was revealed in Samara and Irkutsk regions, as well as in the Republic of Tatarstan (31.9%, 28.7%, and 24.6% of the total number of samples, respectively).The same index for PVM reached a maximum in Nizhni Novgorod, Tver’, and Moscow regions, as well as in the Republic of Tatarstan (11.7%, 10.7%, 10.1%, and 10.5%, respectively); finally, in the case of PVY^NTN^, the maximum level of its presence was registered in the Tver’ and Leningrad regions (8.9% and 8.4%, respectively).

**Figure 4:**
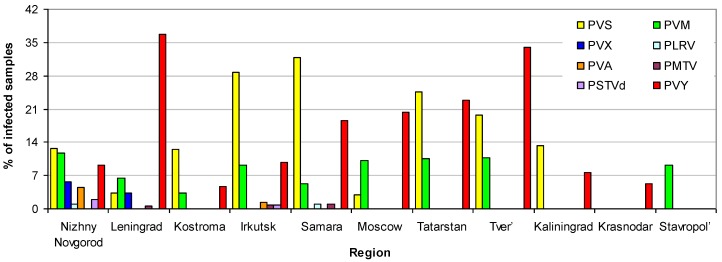
Frequency of the target RNA-based pathogens of potato in the analyzed regions. PVY bars indicate the summed frequency of PVY^O^, PVY^NTN^, and PVY^total^.

The maximum diversity of RNA-based infections was observed in Nizhny Novgorod (PSTVd and seven viruses including both PVY forms) and Irkutsk (PSTVd and six viruses including both PVY forms), as well as in Leningrad and Samara (six viruses including both PVY forms) regions, while only one virus type was detected in Krasnodar Krai and Stavropol’ Krai (Figure 4). High levels of infection (> 30% of samples) was observed in the Leningrad (40.6%), Tver’ (39.3%), Samara (38.9%), and Irkutsk (32.9%) regions and Republic of Tatarstan (31.6%), whereas the Krasnodar Krai and Stavropol’ Krai did not have potato infestation exceeding 10%.

**Figure 5:**
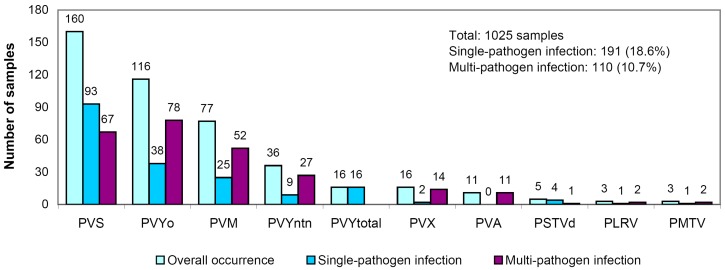
Occurrence of single- and multi-pathogen viral infections in analyzed samples.

The occurrence of different pathogens in infected samples is shown in Figure 5. The most frequent were PVS (detected in 53.1% of infected samples), PVY^O^ (38.5%), PVM (25.6%), and PVY^NTN^ (11.9%); note that the total frequency of PVY (PVY^O^ + PVY^NTN^ + PVY^Total^) was 55.8%, which made this virus the most frequent among other target pathogens. Other pathogens were observed in <6% of infected samples. In relation to the total pool of samples tested, only the first two pathogens were observed with the frequency exceeding 10% (15.6% and 11.3% for PVS and PVY^O^, respectively); in the case of total PVY, this index was 16.4%.

PVS, PVY^O^, PVM, and PVY^NTN^ dominated in the samples with mixed infection, while the rarest species were PMTV, PLRV, and PSTVd. Note that PVA was detected in 11 samples in two regions (predominantly in the Nizhny Novgorod region), always in mixed infections (Table 2, Figure 5).

### 2.3. Mixed Viral Infections

Since the use of PCR micromatrices enables multiplex detection of all the target pathogens, samples with mixed viral infections were also revealed for some regions. Data on the number and composition of samples with mixed viral infections are shown in Table 3. 

No mixed infection was observed in two regions (Krasnodar Krai and Stavropol’ Krai). The maximum number of samples with mixed infection was collected in Nizhny Novgorod, Irkutsk, Samara, and Leningrad regions (20, 19, 18, and 15 samples, respectively), whereas the percentage of samples with mixed infection in the total number of samples was the highest in the Tver’ region and Republic of Tatarstan (19.6% and 19.3%, respectively). In relation to the total number of infected samples, the percentage of mixed infection varied from 22.2% (Kaliningrad region) to 61.1% (Republic of Tatarstan).

In total, we revealed 10 two-pathogen, 8 three-pathogen, and 5 four-pathogen combinations. The maximum number of viral combinations was observed in Nizhny Novgorod (14) and Irkutsk (9) regions; in other cases, 2‒6 combinations were observed. The most frequent combination across the whole set of samples was PVY^O^ + PVS (31 samples) followed by PVM + PVS (17 samples) and PVY^O^ + PVY^NTN^ (13 samples); these three variants composed 55.5% of the total pool of samples with revealed mixed infections.

## 3. Discussion

Successful management of potato diseases requires constant monitoring of the presence of the corresponding pathogens at individual locations of cultivation and in seed material to have a comprehensive understanding of the current situation and to assess the prospects of the diseases’ development and spread. This is especially important for viruses and bacteria, for which no control measures exist after the first manifestation of the symptoms. For some viruses transmitted by insects, such monitoring may provide timely insecticidal treatments of the infected and neighboring fields to prevent the spread of pathogens from infection nodes. Finally, detection of quarantine and regulated pathogens may result in a scheduling of infected fields for several years.

As for bacterial potato pathogens and their occurrence in Russia, there are only two reports describing the distribution of several target pathogens in the period of 2009–2013 [17,18]. It was reported that the IFA analysis of 430 infected potato specimens collected mainly in the central regions of the European part of Russia in 2008–2010 revealed a 3.6% average incidence of *D. dianthicola* and *D. solani* [17]. Re-examination of the same regions in 2013 showed an increase in the level of infection with these pathogens up to 4–36% depending on the region, which significantly exceeded the level of *P. carotovorum* incidence (2–15%). It was reported also that there was evidence of a significant presence of *C. michiganensis* subsp. *sepedonicus* (23–50%) in Russian seed potatoes, as well as *R. solanacearum* likely occurred in the southern regions of Russia [18]. The results obtained with the present study contribute toward a complete analysis of oomycetal, bacterial, and viral pathogens’ distribution in different regions of Russia. We observed that the occurrence of *P. atrosepticum* and *C. michiganensis* subsp. *sepedonicus* in the infected potato for all regions was between 28% and 29.5% that was in agreement with a previous study [17]. At the same time, the occurrence of both *Dikeya* pathogens in the samples collected in 2015–2018 (0.3% and 3.8% of the total and infected samples, respectively) were reduced with respect to the previous report conducted in 2014 (up to 36%) [17]. This fact may probably be explained by a shift in the composition of pathogens causing black leg in potato reported in 2017 [34]. According to this study, the analysis of several hundreds of potato samples from different regions of Russia performed in autumn of 2017 showed a complete disappearance of *Dickeya* spp. and a significant reduction of the occurrence of *P. atrosepticum* and *P. carotovorum* subsp. *carotovorum* (4% and 18%, respectively); in the same study, a significant infection of analyzed samples with other potato pathogens, *P. wasabiae* and *P. carotovorum* subsp. *brasiliensis* was reported (12% and 49%, respectively).

In the case of viral potato pathogens, information about the current situation in Russia is very limited and mainly describes the situation in separate regions. For example, field monitoring performed in the Far East in 2005 and 2008 showed the prevalence of PVX and PVM infections, as well as a wide occurrence of PSTVd [12]. Recent examination of potato fields in the northwestern regions of Russia showed a high level of infection with PVY (71%), also in concomitance with PVM (7%) [14]. Authors also reported the prevalence of PVY (65–95%) in some territories of the Astrakhan region (southern Russia) with single infections with PVS and PVM. Finally, the monitoring of the PVY occurrence in different regions of Russia performed in 2013 confirmed the predominance of this virus in the northeastern regions of Central Russia, Volga region, and the Tomsk region (65–85%) [16]. Results of our study confirmed the earlier reported data on the dominance of PVY in most regions of central and northwestern Russia and provided a lot of additional information about the occurrence of other viruses and the level and composition of viral infections in the studied regions, which, along with the similar data on bacterial pathogens, can be useful for potato growers of these regions in relation to the choice of resistant cultivars and potato management strategies based on the known epidemiological situation. 

The performed survey allowed us to obtain a large volume of relevant information in relation to the 15 target DNA- and RNA-based pathogens. In this aspect, our study is the first large-scale survey of a wide range of potato pathogens in Russian regions. The studies describing the occurrence of more than five potato pathogens include the surveys of five potato viruses in Tunisia (PVA, PVX, PVY, PVS, and PLRV; [27]) and China (PVA, PVX, PVY, PVM, and PLRV; [26]), and six potato viruses (PVX, PVY, PVS, PVM, PVA, and PLRV) in Pakistan [28] and China [25]. A possibility of simultaneous identification of multiple pathogens in the same sample provided us with very interesting and valuable information about the composition of mixed infections and the frequency of various combinations. For the “bacterial” part of the study, the number of samples with revealed mixed infection was too low to make any conclusions; at the same time, the “viral” part of the study provided a significant number of such samples. The surveys performed in Pakistan [28] and China [26] also provided some information about the frequency and composition of mixed viral infections. The most frequent viruses in mixed infections observed with the present study were PVY, PVS, and PVM (95.5%, 60.9%, and 47.3%, respectively), whereas in China and Pakistan they were PVY, PLRV, PVX, and PVM (80.0%, 66.7%, 46.7%, and 23.3%); and PVY, PVX, PVS, and PVM (53.9%, 49.4%, 36.4%, and 20.5%), respectively. The number of viral combinations revealed in our study (23 combinations) significantly exceeded those reported in the mentioned studies (12 and 7 combinations for References [28] and [26], respectively), and the most frequent variant in Russia (PVY+PVS, 32.7% of the total number of samples with a mixed infection) was reported only for Pakistan (14.3%). The data on the high level of PVS presence in mixed viral infections in Russia are very important, since this virus, being symptom-free alone in the majority of cultivars, induces severe co-infection with PVY, PVX, and PVA [28].

Thus, our study is the first large-scale screening of a wide range of potato pathogens in Russian regions located in different climatic zones, conducted in a network mode using a unified methodology and standardized qPCR micromatrices. It provides detailed information on the phytosanitary status of selected regions and the geographical spread of individual pathogens, and is the first one to give detailed information on the presence of mixed infections, including their composition, in potato fields of Russia. The results of this survey will most likely be used for further harmonization of seed certification in the Russian Federation [35] with international requirements.

## 4. Materials and Methods

### 4.1. Survey Arrangement

**Figure 6:**
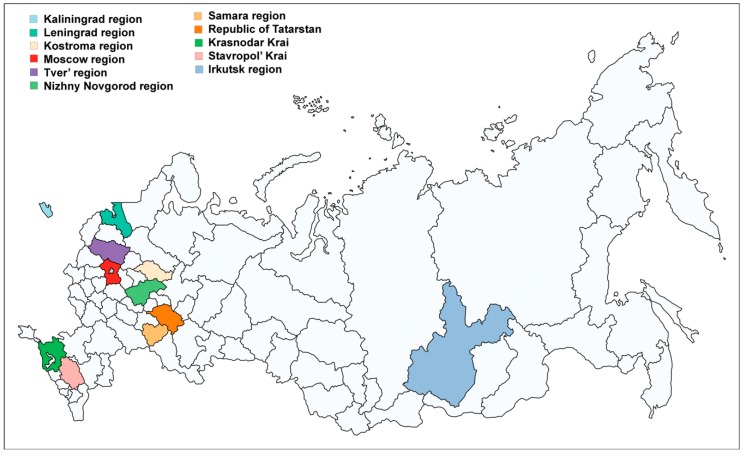
Regions of Russia where the survey was arranged.

The study was carried out in 2015–2018 on the basis of 11 regional branches of the Russian Agricultural Center (RAC) in Kaliningrad, Leningrad, Tver’, Moscow, Kostroma, Nizhny Novhorod, Irkutsk regions, Stavropol’ Krai, Krasnodar Krai, and the Republic of Tatarstan, and at the private potato producing company from the Samara region (Figure 6). 

Potato samples (leaves for detection of viral infections and tubers for detection of bacterial/oomycetal infections) were collected from commercial fields located in the corresponding regions in accordance to the standard recommendations [36] in the spring–autumn period. The total number of leaf and tuber samples was 1025 and 725, respectively. Samples were analyzed in field laboratories of the corresponding regional RAC branches except for the samples from Krasnodar region, which were treated at the Moscow regional RAC branch. The analysis of each sample was repeated 3‒5 times.

According to preceding laboratory trials [33] and at the initial stage of this study, all the results obtained with PCR matrices were compared with results obtained with the conventional techniques (ELISA, LFD, microbiological assessment) on the same specimens. 

### 4.2. Configuration and Characteristics of PCR Micromatrices

The technology of stationary PCR micromatrices with open reactors provides for a flexible configuration with the possibility to vary the set of target pathogens according to customer’s needs. In this study, two types of ready-to-use PCR micromatrices were used developed by GenBit LLC in collaboration with the All-Russian Research Institute of Phytopathology [33]:

- RT-PCR micromatrix “Potato pathogens. RNA” was used for simultaneous detection and identification of specific RNA sequences of PVY (PVY^O^ and PVY^NTN^ forms), PVX, PVA, PVS, PVM, PLRV, and PMTV, as well as PSTVd. The matrix design provides for simultaneous examination of two samples per matrix.
- PCR micromatrix “Potato pathogens. DNA” was used for simultaneous detection and identification of specific DNA sequences of *Phytophthora infestans*, *Pectobacterium atrosepticum*, *P. carotovorum* subsp. *carotovorum*, *Dickeya dianthicola*, *D. solani*, *Clavibacter michiganensis* subsp. *sepedonicus*, and *Ralstonia solanacearum*. The matrix design provides for simultaneous examination of three samples per matrix.

The primers and probes for the above-mentioned test systems were reported in a previous study [33]. Since the amplification of all test systems arranged in the same micromatrix should occur simultaneously, these oligonucleotides were specially designed to meet the “standard” amplification parameters. Each micromatrix contained an internal control (IC) for each sample, and also positive and negative controls for each pathogen included. Laboratory studies of the test systems and composed micromatrices demonstrated the absence of any cross-reactions or false positive results [33]. The detection limit for all pathogens was 1 ng/mL except for general PVY (0.1 ng/mL).

### 4.3. DNA/RNA Extraction and Sample Application

DNA and RNA extraction was carried out using an AmpliSens® DNA-sorb-B DNA extraction kit and RIBO-sorb kit for DNA/RNA extraction (The Central Research Institute of Epidemiology, Moscow, Russia) according to the manufacturer’s recommendations. DNA samples were stored at −20°C until use. After installation of the corresponding ready-to-use micromatrix into a holder, the whole reaction zone was completely covered with a sealing layer of mineral oil (620 μL), avoiding bubble formation. DNA or RNA samples were mixed with a 10× PCR buffer (SibEnzyme, Novosibirsk, Russia) at a 1:9 ratio. One microliter of a resulted sample or deionized water (negative control) was added into each well under the sealing oil layer according to a particular matrix topology. 

### 4.4. Amplification in Micro-matrix Format and Data Analysis

Real-time PCR/RT-PCR was performed using a two-beam AriaDNA® Microchip Amplifier (Lumex-Marketing LLC, St.-Petersburg, Russia). Standard thermal cycling conditions included initial denaturation (94 °C for 120 s) followed by 45 cycles of 94°C for 5 s and 60 °C for 25 s. For RNA samples, the process included a preliminary reverse transcription stage (37 °C for 20 min). Detection of fluorescence related to PSTVd, PVYo, PVS, PMTV, PVA, *C. michiganensis* subsp. *sepedonicus*, *P. atrosepticum*, *P. carotovorum* subsp. *carotovorum*, and *D. solani* was followed using channel 1 (FAM). For PLRV, PVYNTN, PVX, *P. infestans*, *D. dianthicola*, *R. solanacearum*, and the internal control (IC), the channel 2 (ROX) was used. Signal recording, calculation of threshold cycles (Ct), and analysis of the results were performed automatically using an AriaDNA software package (Lumex-Marketing LLC., Russia). Statistical treatment of data was carried out using an MS Excel 2003 program package.

## Figures and Tables

**Table 1:** Results of potato testing for bacterial/oomycetal infections with the use of “Potato pathogens. DNA” diagnostic PCR micromatrices.

Region of Russia	Year of Collection	Number of Samples	Number of Infected Samples	*Pa ^1^*	*Pcc*	*Ds*	*Dd*	*Cms*	*Rs*	*Pi*
Leningrad region	2015–2018	65	12	3	7	0	0	3	0	0
Kostroma region	2015–2018	214	19	3	4	3	0	11	2	2
Moscow region	2017–2018	38	3	2	3	0	0	1	0	0
Tver’ region	2017–2018	62	40	20	14	1	0	12	0	9
Nizhny Novgorod region	2015–2018	141	12	2	2	0	0	0	0	8
Samara region	2017–2018	22	4	0	2	0	0	0	0	2
Republic of Tatarstan	2017–2018	6	2	0	0	0	0	0	0	2
Krasnodar Krai	2017	19	4	0	0	0	0	0	0	4
Stavropol’Krai	2017–2018	7	1	0	1	0	0	0	0	0
Irkutsk region	2015–2018	151	35	7	3	0	1	12	0	17
Total number of samples		725	132	37	36	4	1	39	2	44

^1^ Rs, *Ralstonia solanacearum*; Pa, *Pectobacterium atrosepticum*; Pcc, *P. carotovorum* subsp. *carotovorum*; Cms, *Clavibacter michiganensis* subsp. *sepedonicus*; Pi, *Phytophthora infestans*; Ds, *Dickeya solani*, Dd, *D. dianthicola*.

**Table 2:** Results of potato testing for viral/viroid infections with the use of “Potato pathogens. RNA” and “Potato pathogens. PVY” diagnostic PCR micromatrices.

Region of Russia	Year of Collection	Number of Samples	Number of Infected Samples	PVY^O^	PVY^NTN^	PVS	PVM	PVX	PLRV	PVA	PMTV	PSTVd	PVY^total^
Kaliningrad region	2017–2018	53	9	2	2	7	0	0	0	0	0	0	0
Leningrad region	2015–2018	155	63	28	13	5	10	5	0	0	1	0	16
Kostroma region	2015–2018	152	24	7	0	19	5	0	0	0	0	0	0
Moscow region	2017–2018	69	15	10	4	2	7	0	0	0	0	0	0
Tver’ region	2017–2018	56	22	14	5	11	6	0	0	0	0	0	0
Nizhny Novgorod region	2015–2018	197	57	17	1	25	23	11	2	9	0	4	0
Samara region	2017–2018	113	44	17	4	36	6	0	1	0	1	0	0
Republic of Tatarstan	2015–2018	57	18	11	2	14	6	0	0	0	0	0	0
Krasnodar Krai	2017	19	1	0	1	0	0	0	0	0	0	0	0
Stavropol’Krai	2017–2018	11	1	0	0	0	1	0	0	0	0	0	0
Irkutsk region	2015–2018	143	47	10	4	41	13	0	0	2	1	1	0
Total number of samples		1025	301	116	36	160	77	16	3	11	3	5	16

**Table 3:** Number of samples with mixed viral infections and their viral composition.

Region	Combinations of Viruses	Number of Samples	% of Mixed Infection in the Total Number of Samples	% of Mixed Infection in the Total Number of Infected Samples
Leningrad region	PVY^O^ + PVY^NTN^	7	9.7	23.8
	PVY^O^ + PVM	4		
	PVY^O^ + PVX	2		
	PVY^O^ + PVX + PMTV	1		
	PVX+ PVS+ PVM	1		
Kostroma region	PVY^O^ + PVM	1	4.6	29.2
	PVY^O^ + PVS	5		
	PVM + PVS	1		
Moscow region	PVY^O^ +PVM	1	10.1	46.67
	PVY^O^+ PV^YNTN^	3		
	PVY^O^+ PVY^NTN^ +PVM	1		
	PVM + PVS	2		
Tver’ region	PVY^O^+ PVY^NTN^ + PVS	1	19.6	50.0
	PVY^O^+ PVY^NTN^	3		
	PVY^O^ + PVS	2		
	PVY^O^ + PVM	2		
	PVY^O^ + PVS + PVM	2		
	PVY^NTN^ + PVS	1		
Nizhny Novgorod region	PVY^O^ + PVS	1	10.2	35.1
	PVY^O^ + PVM	1		
	PVY^O^ + PVX	1		
	PVM + PVS	2		
	PVM + PVA	1		
	PVM + PVX	2		
	PVM + PSTVd	1		
	PVM + PVX + PVA	2		
	PVY^O^ + PVM + PVA	2		
	PVY^O^ + PVM + PVX	2		
	PVY^O^+ PVY^NTN^ + PVS	1		
	PVY^O^ + PVX + PVA + PLRV	1		
	PVY^O^ + PVM + PVX + PVA	2		
	PVY^O^ + PVM + PVA + PLRV	1		
Irkutsk region	PVY^O^ + PVS	5	13.3	40.43
	PVY^O^ + PVM + PVS	1		
	PVY^O^ + PVY^NTN^ + PVS	1		
	PVY^O^ + PVY^NTN^ + PVS + PVM	1		
	PVY^NTN^ + PVS	1		
	PVY^NTN^ + PVS + PVM + PMTV	1		
	PVM + PVS	7		
	PVM + PVA	1		
	PVS + PVA	1		
Samara region	PVY^O^ + PVS	11	15.9	40.9
	PVY^O^ + PVM +PVS	2		
	PVY^O^ + PVY^NTN^ + PVM	1		
	PVY^NTN^ + PVS	2		
	PVM + PVS	2		
Republic of Tatarstan	PVY^O^ + PVS	6	19.3	61.1
	PVY^O^ + PVY^NTN^ + PVS + PVM	2		
	PVM + PVS	3		
Kaliningrad region	PVY^O^ + PVS	1	3.8	22.2
	PVY^NTN^ + PVS	1		
